# Supplementary material for: Altered bioenergetics and mitochondrial dysfunction of monocytes in patients with COVID‐19 pneumonia
Source: EMBO Mol Med. 2020 Nov 5;12(12):e13001. doi: 10.15252/emmm.202013001 (PMC7645870; doi:10.15252/emmm.202013001)
Supplement: Supplementary file 2 — Appendix [file EMMM-12-e13001-s002.pdf]

EMBO Molecular Medicine

Altered bioenergetics and mitochondrial dysfunction of monocytes in patients with COVID-19 pneumonia

Lara Gibellini, Sara De Biasi, Annamaria Paolini, Rebecca Borella, Federica Boraldi, Marco Mattioli, Domenico Lo Tartaro, Lucia FIdanza, Alfredo Caro-Maldonado, Marianna Meschiari, Vittorio Iadisernia, Erica Bacca, Giovanni Riva, Luca Cicchetti, Daniela Quaglino, Giovanni Guaraldi, Stefano Busani, Massimo Girardis, Cristina Mussini, and Andrea Cossarizza
DOI: 10.15252/emmm.202013001

Corresponding authors: Lara Gibellini (lara.gibellini@unimore.it) , Andrea Cossarizza (andrea.cossarizza@unimore.it)

Review Timeline:

Submission Date:	25th Jun 20
Editorial Decision:	15th Jul 20
Revision Received:	31st Jul 20
Editorial Decision:	10th Aug 20
Authors' Correspondence:	10th Aug 20
Editor's Correspondence:	10th Aug 20
Revision Received:	14th Sep 20
Editorial Decision:	7th Oct 20
Revision Received:	9th Oct 20
Accepted:	14th Oct 20

Editor: Celine Carret / Zeljko Durdevic

Transaction Report:

(Note: With the exception of the correction of typographical or spelling errors that could be a source of ambiguity, letters and reports are not edited. Depending on transfer agreements, referee reports obtained elsewhere may or may not be included in this compilation. Referee reports are anonymous unless the Referee chooses to sign their reports.)

15th Jul 2020

Decision on your manuscript EMM-2020-13001

Dear Dr. Gibellini,

Thank you for the submission of your manuscript "Metabolic exhaustion of monocytes in COVID-19 patients". We have now heard back from the three referees whom we asked to evaluate your manuscript.

As you will see, while ref. #3 is supportive of publication, ref. #2 and more so ref. #1 have serious reservations about the manuscript. I will not get into details here, but the lack of clear significance and relevance of the findings as more than correlations, along with incomplete data analyses in several parts and technical limitations weaken the study to a point where our one round of main revision would not be enough to make the paper suitable for publication in EMBO Molecular Medicine.

We therefore prefer to reject now in order to avoid further frustration down the line and allow you to resubmit quickly somewhere else. I am sorry to have to disappoint you on this occasion and hope that the referee comments will be helpful in your continued work in this area.

***** Reviewer's comments *****

Referee #1 (Remarks for Author):

In this study entitled "Metabolic Exhaustion of Monocytes in Covid-19 patients", Gibellini et al first assessed metabolic properties of total monocytes from COVID-19 patients, described monocyte subset proportion in COVID-19 patients by flow cytometry as well as expression of inhibitory checkpoints in such monocyte subsets. They report that 1) total monocytes from COVID-19 patients exhibit metabolic impairment, with decreased OXPHOS and impaired oxidative burst, 2) changes in the distribution of monocyte subsets, with an expansion of intermediate monocytes and lastly 3) increase in inhibitory receptors, including PD-1 and PD-L1 in all subsets.

This is a highly descriptive study with no clear relevance. In addition, experiments are not performed to the highest standards (flow cytometry gating is questionable) or designed correctly (Why measuring metabolic activity of total monocytes if the authors found an expansion of intermediate monocytes in COVID-19 patients?). I also do not understand the connection between metabolic exhaustion and PD-1/PD-L1 expression? Same to conclude on the exhausted nature of a cell

because of expression of PD-1/PD-L1......expression of PD-1/PD-L1 could simply reflect the suppressive nature of the COVID-19 patient environment... and not any exhaustion. In addition, it seems that the manuscript was written in a rush as not easily readable and the figures are poorly designed (no panel to make the figures clearer).

The flow cytometry strategy to identify monocyte subsets is not optimal in the COVID-19 patients and I believe that there is a clear overestimation of intermediate monocytes by misplacing the gate to identify them. Furthermore, gating on HLA-DR+ cells limits the analysis of monocytes considering non-classical monocytes express low levels of HLA-DR. Monocytes in critically ill patients may have reduced HLA-DR expression on their monocytes. The authors do not investigate this possibility by their gating strategy. By redesigning their gating approach, it could provide information about the phenotype of these cells. There was no isotype control used for any of the histograms which brings into question the validity of PD-1 and PD-L1 expression. This is essential for flow cytometry representation and could be added to the appropriate figures.

The term "metabolic exhaustion" is not supported by the authors findings. The limited metabolic response seen by the authors could be caused by a number of factors such as IL-10 signalling or immature monocyte release (https://science.sciencemag.org/content/356/6337/513/tab-figures-data and https://www.nature.com/articles/s41467-018-07215-9). Neither of these possibilities are ruled out by the experiments presented in this paper.

Supporting information regarding metabolic observation is practically devoid from any experimental observations made by the authors. The message of the article should be changed to match the observations made in the article.

The authors claim that the monocytes have less ability to maintain maximal respiration; however, the maximal respiration is never the same, and the loss of looks similar to controls. This claim needs to be modified, or appropriate comparisons supporting this claim should be presented.

Claiming that classical monocytes "only tended to decrease" is not supported by statistical analysis and therefore the claim needs to reflect that.

The citation Riva and Mehta states that monocyte expression of PD-1, PD-L1 and TIM-3 are involved in immune suppression, but the link to sepsis is missing. The authors need to specifically identify the source of the claim "in general, expression of inhibitory immune checkpoints, like PD-1, PD-L1 and TIM-3 has been linked to a more suppressive phenotype and worse prognosis in sepsis, infections and even cancer. If co-inhibitory receptor/ligand expression is linked to immune suppression, why is sepsis more likely in these patients?

Age and population demographics of control cohort is missing and brings into question whether the populations are comparable.

Does disease severity impact monocytes differently? The authors group all covid-19 patients together, but do not show disease severity stratification.

The introduction of PD-1 expression and ligand expression is missing a citation.

Referee #2 (Comments on Novelty/Model System for Author):

In their manuscript "Metabolic exhaustion of monocytes in COVID-19 patients" the authors address COVID-19 associated changes in monocyte subset distribution, monocyte phenotype and metabolism. With their study the authors aim at elucidating mechanisms of hyper-inflammation observed in COVID-19 that are associated to monocytes. As hyper-inflammation is a complication of COVID-19 associated with severe disease and at the same time displays an attractive target for therapeutic intervention on different levels, a detailed understanding of the underlying mechanisms is urgently needed. While this is an important clinical aspect with implications also e.g. for treatment with immune checkpoint inhibitors, only very little is known about modulation of PD-1 and PDL-1 during SARS-CoV-2 infection. Therefore the Topic of the manuscript is of high novelty and potential Impact.
In their study, the authors analyzed monocyte metabolic parameters and monocyte subset distribution as well as monocyte expression of PD-1, PDL-1 and TIM-3 in COVID-19 patients and healthy controls. While novelty, medical impact and adequacy of the system are rated high, technical quality is rated medium due to lack of information on the status of the SARS-CoV-2 infection of the patients at the time-point of sampling, i.e. in which phase of COVID-19 they were, and lack of information which of the 15 patients were analyzed for monocyte subsets and marker expression (see specific comments to the authors below).

Referee #2 (Remarks for Author):

Major comments to be addressed during revision:
1) The authors analyzed 15 COVID-19 patients and 12 healthy controls which correspond to the data shown in figure 1. Scatter plots in figure 3 and 4 seem to show fewer data points and information on which patients were analyzed and why not all patients were analyzed is lacking.
2) While longitudinal analyses for more time-points would be optimal but are not always feasible or possible, more information on the patients' diseas and inflammatory status at the time-point of analysis would facilitate integration and interpretation of the results. Relevant information would be whether these patients were altogether in a similar stage of the disease, what time-point from diagnosis/onset of the symptoms/positive test the samples were taken or whether they had already developed a specific adaptive immune/antibody response.
3) The interpretation of the clinical relevance of the presented findings would be greatly increased, if there are correlations to inflammatory parameters. If the authors have analyzed, or have a chance to analyze systemic cytokine levels such as for IL-6 for the time-point of monocyte analysis, this should be shown or done. As is stated also by the authors, it is a limitation that cytokine production by the monocytes themselves could not be analyzed - this is the case especially as monocyte metabolic exhaustion seems somewhat counter-intuitive in hyper-inflammation. Also therefore, any correlation to the disease and inflammatory status of the patients at the time-point of analysis would be very interesting and helpful.
4) According to figure 2, total monocytes and monocyte subsets were analyzed for the expression of PDL-1, TIM-3, CXCR3, CCR2 and CD38. These data are however shown only for PD-1 and PDL-1 and described for TIM-3. The description of the TIM-3 data should include "data not shown" while CXCR3, CCR2 and CD38 analyses need not to be mentioned in the text and shown in the gating strategy in figure 2, if the results of these analyses are not shown, described or discussed later in the manuscript or supplement.

Minor comments to be addressed during revision:
1) Organization of multi-panel figures into A, B etc. would facilitate reading and finding the relevant (sub)-figure.
2) Please check scatter-plots in figure 1 as Max Resp maintenance is listed in the figure legend but

Basal ECAR is shown (and not listed in the legend).
3) Are those few patients showing substantially higher values in the bioenergetics profile (figure 1) always the same patients and does this correlate with any other parameter of the disease (along major comments 2) and 3))?
4) In the gating strategy (figure 2) the description is a bit misleading regarding CD15/CD11b gating. The text states "Then, cells positive for HLA-DR and CD15 (i.e., leukocytes that did not include lymphocytes, that are CD15-) were selected" and CD11b is not referred to, while in the corresponding plot the y-axis is labelled CD15/CD11b and there does not seem to be a selection for the CD15/CD11b-positive population but rather all HLA-DR positive cells are gated.

General comments (not necessarily need to be addressed for publication but of general interest):
1) As this manuscript clearly focusses in monocytes, also T cell PD1/PD-L1 expression would be of high interest.
2) It would be interesting to comment or speculate on the relevance of these results on a treatment with check-point inhibitors in cancer patients (during concomitant COVID-19).

Referee #3 (Remarks for Author):

This is a very interesting manuscript which characterizes functional changes in peripheral blood monocytes from patients affected by Covid 19 pneumonia. The Authors identified a redistribution among different monocytes classes accompanied by a significant expansion of intermediate/pro-inflammatory cells and an increased expression of inhibitory checkpoints, including PD-1/PD-L1. It turns out that the metabolic and functional exhaustion in monocytes from patients might alter their capability to rapidly clear the infection. The manuscript is concise, well written and unveils novel aspects of Covid 19 infection, not characterized before. I suggest that some points are addressed before publication, including experiments, if the limited material from patient samples allows to perform them:

Major points
1. In Fig. 1, left panel with representative traces, it is not clear to me if cells from Covid patients contain less mitochondria than control monocytes or if their mitochondria are simply less functional. A difference in number of mitochondria might explain a different basal and maximal respiration. Cardiolipin staining with Nonyl Acridine Orange (NAO) fluorescent mitochondrial dye whose staining is independent of mitochondrial membrane potential, might help to characterize the mechanism causing lower respiration in cells derived from Covid patients.
2. Western blotting of respiratory complex subunits, ATP synthase and its inhibitor protein IF1 should be performed, if lysates from patient cells are available. On one hand OXPHOS detection will provide a possible explanation for the functional results presented in Fig. 1, on the other hand, the IF1 levels would be particularly interesting given the emerging role of this protein in the modulation of ATP synthase in pathological condition and inflammation (García-Aguilar A and Cuezva JM, Front Physiol 2018. Review of the Inhibition of the Mitochondrial ATP Synthase by IF1 in vivo: Reprogramming Energy Metabolism and Inducing Mitohormesis; Satapati S et al., J Clin Invest 2015. Mitochondrial metabolism mediates oxidative stress and inflammation in fatty liver).

Minor points
1. I suggest to the Authors to briefly mention in the introduction the connection between monocyte ATP level (bioenergetic performance/glycolysis) and their role in the infection clearance. This would help the less specialized audience to follow the importance of the presented data and understand

the relevance of differences in the respiratory behavior of patient cells.
2. In Supplementary Table 1, a range of values in normal condition (healthy donors) for bilirubin, LDH, and other parameters (that are reported in the text) should be indicated on the right in the same table, for clarity.
3. The "oxidative burst" which is stimulated by ionomycin treatment should be better explained in the text. This treatment may cause multiple effects such as Ca2+ uptake in mitochondria, stimulation of the respiratory chain, depolarization of the mitochondrial membranes, ... The Author should better explain which of these effects are in their opinion emerging in their experimental conditions and help the reader to understand the importance of differences between patient and control monocytes.

Dear Dr Carret,

We are sorry to disturb you. A few days ago we submitted a manuscript entitled "Metabolic exhaustion of monocytes in COVID-19 patients" to be considered for publication in EMBO Molecular Medicine.

The manuscript was rejected after the evaluation by three reviewers. The overall evaluation of two of them (referee #2 and #3) was fairly good as novelty, medical impact and adequacy of the system were rated high. The main general weaknesses were the lack of due to lack of information on the status of COVID-19 disease of the patients at the time-point of sampling and the characterization of monocytes by flow cytometry. These comments are actually quite easy to address.

We are writing you because we present a rebuttal, as we do not agree with criticism made by referee #1, who made questionable comments and seems not so competent in the field of flow cytometry. This is quite disappointing, as a relevant part of the manuscript is based on data obtained with this technique.

We are thus sending a file, where you can find our point-to-point answers to the observations of referee #1, together with answers to referees #2 and #3.

We have partially re-thought the structure of the manuscript, which could obviously include the requests of the referee #2 and #3, and also new data such as the unsupervised analysis of flow cytometry data (showing that our gating strategy was not wrong at all, as stated by referee #1), electron micrographs showing ultrastructural alterations in mitochondria, and in vitro stimulation of monocytes (that produce TNF and IFN-gamma, in spite of their clear alterations).

For these reasons, we do hope you can accept our comments about the revision and our rebuttal, and can consider again our work for EMBO Molecular Medicine. We thank you in advance for your time.

Looking forward to hearing from you,

Sincerely yours,

Andrea Cossarizza

Lara Gibellini

2nd Editorial Decision 10th Aug 2020

10th Aug 2020

Dear Dr. Gibellini,

Thank you for asking us to reconsider our decision on this article and apologies for only getting back to you today. I decided to seek advice on your article and consulted with our chief editor and an expert in the field.

I am happy to convey that we would like to invite revision of the paper according to your rebuttal letter.

Our advisors say "establishing a causative link is impossible in patients, hence the best would be to consolidate the immunological "environment" of these monocytes and further approach the heart of the process by using monocytes purified from alveolar lavages [...]". We would therefore strongly encourage you to make the study stronger by providing additional data as much as you possibly can and re-analysis as recommended.

Please keep in mind that the referees' concerns must be fully addressed and that acceptance of the manuscript would entail a second round of review.

EMBO Molecular Medicine has a "scooping protection" policy, whereby similar findings that are published by others during review or revision are not a criterion for rejection. Should you decide to submit a revised version, I do ask that you get in touch after three months if you have not completed it, to update us on the status.

Please also contact us as soon as possible if similar work is published elsewhere. If other work is published we may not be able to extend the revision period beyond three months.

Please read below for important editorial formatting and consult our author's guidelines for proper formatting of your revised article for EMBO Molecular Medicine.

I look forward to receiving your revised manuscript.

Authors' Correspondence 10th Aug 2020

Dear Dr. Carret,

Thank you for the message and the information.
There one unsolvable problem with the note of your advisors. According to international guidelines, BAL can be done only for clarifying a clinical suspect of COVID pneumonia in a patient with a negative swab. In fact, BAL poses at high risk the operators. In our clinics, only 3 BAL have been done in 3 months, in a population of more than 300 patients. Fortunately, in the last weeks we have seen just a couple of pneumonia. So, studying BAL now is almost impossible for us.
Thus, I am asking you if the paper will be considered even without data from BAL.
Thank you in advance for your consideration.
Kind regards,
Andrea Cossarizza and Lara Gibellini.

Editor's Correspondence 10th Aug 2020

Dear Drs. Cossarizza and Gibellini,

I apologize for the brevity of my letter earlier today. Our advisor indeed mentioned BAL to start with but added that it would be very difficult if not impossible to perform given the circumstances. I'm sorry I should have added this bit to the letter.

So yes, to answer your question, we would still consider the paper even if you don't perform BAL.
Looking forward to receiving the revision.

Point-to-point responses to reviewers' comments

Referee #1

In this study entitled "Metabolic Exhaustion of Monocytes in Covid-19 patients", Gibellini et al first assessed metabolic properties of total monocytes from COVID-19 patients, described monocyte subset proportion in COVID-19 patients by flow cytometry as well as expression of inhibitory checkpoints in such monocyte subsets. They report that 1) total monocytes from COVID-19 patients exhibit metabolic impairment, with decreased OXPHOS and impaired oxidative burst, 2) changes in the distribution of monocyte subsets, with an expansion of intermediate monocytes and lastly 3) increase in inhibitory receptors, including PD-1 and PD-L1 in all subsets.

This is a highly descriptive study with no clear relevance. In addition, experiments are not performed to the highest standards (flow cytometry gating is questionable) or designed correctly (Why measuring metabolic activity of total monocytes if the authors found an expansion of intermediate monocytes in COVID-19 patients?). I also do not understand the connection between metabolic exhaustion and PD-1/PD-L1 expression? Same to conclude on the exhausted nature of a cell because of expression of PD-1/PD-L1......expression of PD-1/PD-L1 could simply reflect the suppressive nature of the COVID-19 patient environment... and not any exhaustion. In addition, it seems that the manuscript was written in a rush as not easily readable and the figures are poorly designed (no panel to make the figures clearer).

Authors' answer: We did not draw conclusions on the exhausted nature of monocytes just because of expression of PD-1/PD-L1, but considering all data reported in this manuscript, *i.e.* reduced OCR, reduced ECAR, reduced oxidative burst, expression of inhibitory receptors. Moreover, the expression of PD-1/PD-L1 could not simply reflect the suppressive nature of the COVID-19 patient environment as the patients' environment is both suppressive and hyperinflammatory, as reported by De Biasi et al., Nature Comm (2020) and others (Mehta et al., The Lancet, 2020; Ragab et al., Front Immunol, 2020). However, we improved the text of the manuscript and rearranged the figures, and part of the data was moved to the Supplementary Material. New figures comprise data regarding monocyte bioenergetics, electron micrographs describing ultrastructural changes characterizing monocytes from COVID-19 and CTR, unsupervised analysis of flow cytometry data, quantification of immature monocytes, and quantification of plasma level of cytokines and chemokines involved in monocyte regulation.

The flow cytometry strategy to identify monocyte subsets is not optimal in the COVID-19 patients and I believe that there is a clear overestimation of intermediate monocytes by misplacing the gate to identify them. Furthermore, gating on HLA-DR+ cells limits the analysis of monocytes considering non-classical monocytes express low levels of HLA-DR. Monocytes in critically ill patients may have reduced HLA-DR expression on their monocytes. The authors do not investigate this possibility by their gating strategy. By

redesigning their gating approach, it could provide information about the phenotype of these cells. There was no isotype control used for any of the histograms which brings into question the validity of PD-1 and PD-L1 expression. This is essential for flow cytometry representation and could be added to the appropriate figures.

Authors' answer: The goal of gating strategy is to identify cells on the basis of the expression of different markers. This is operator dependent and it is performed by following well-established rules, such as the use of Fluorescent Minus One controls (FMO) in which all the antibodies are mixed in the tube except of one per time. This allows performing two different steps: to check if the compensation (performed by single stained controls) is correct and to set a gate for the missing antibody. Moreover, the gating strategy should be checked on healthy subjects and pathological sample in order to establish a gating strategy that could be as impartial as possible. Moving the gate in a different way if the analysis is performed on healthy donors and patients is not a good method and not included in any standard operating procedures. Hence, to reply to the referee's comment "*I believe that there is a clear overestimation of intermediate monocytes by misplacing the gate to identify them"*, there is no overestimation of intermediate monocytes as there is an increase in CD16 MFI fluorescence that implies an increase in the percentage of intermediate monocytes.

We are well aware that even following good rules, setting gates is in any case operator dependent and someone considers this an "art". For these reasons, we applied an unsupervised approach to analyze monocytes subpopulations in CTR and COVID-19 patients. Multiparametric unsupervised method, such as the combination of different algorithms of automatic clustering (FlowSOM), dimension reduction representation (UMAP) and statistical methods for differential discovery analyses in high-dimensional cytometry data (diffcyt) are mostly automatic and operator independent. A new Figure could show the results of this analysis, confirming those obtained by manual gating. So, again, COVID-19 patients are characterized by lower percentage of classical monocytes and higher percentage of intermediate monocytes. The manual gating strategy together with the quantification of monocytes and their subsets by manual gating has been moved in supplementary materials.

Regarding the expression of HLA-DR, we are well aware that the number of molecules expressed by intermediate and non-classical monocytes are low, and in particular, in pathological conditions such as sepsis, the dysfunctional monocytes downregulate the expression of HLA-DR. For these reasons, we included the HLA-DR dim population in the gate of CD14 in each samples (as shown in gating strategy). Moreover, CD14-APC mAb has been titrated (like all the other antibodies) and the separating and saturating titer was always used. Thus, the data reported in the paper has been analyzed according to reviewer's suggestions.

Concerning the use of isotype controls we do not agree with the comments for several reasons, that have been discussed several times in the last 15-20 years, especially in the Purdue List (see http://www.cyto.purdue.edu/hmarchiv/index.htm), where the most renown experts of flow cytometry share

basic and most complex notions regarding flow cytometry technique and data analyses (both fluorescent and mass cytometry). The main limitations and uselessness of isotype controls are briefly reported here below:

1) Individual antibody conjugates have various levels of background staining, depending upon their specificity, concentration, degree of aggregation and fluorophore:antibody ratio. Hence the hit-or-miss prospect to find an isotype that truly matches the background staining of a particular test antibody. Isotype controls do not define the true level of background staining and the use of these creates a circular position.
2) Isotype controls do not account for fluorescence spillover from other channels. Only for this, the are in fact useless when several fluorescences are simultaneously used.
3) Much more information is contained in the exhaustive note that was written by Dr. Mario Roederer from NIH (universally recognized as the top expert in this field) in 1998, regarding the use of isotype controls (https://lists.purdue.edu/pipermail/cytometry/1998-April/009860.html).

The appropriate figure is now reported in the paper: the MFI shift between CTR and COVID samples clearly shows that there is an increased expression of PD-L1 in COVID monocytes.

The metabolic activity was evaluated on total monocytes because our primary endpoint was to find metabolic impairment between monocytes from COVID patients and healthy donors. We discovered that monocytes from COVID patients display low oxidative burst if compared to healthy donors. For this reason, in order to ascertain if this difference could be due to a different monocyte distribution, monocyte phenotype was evaluated. We tried to sort intermediate monocyte from COVID patients, but the number of cells obtained was not sufficient to perform further metabolic analyses.

The term "metabolic exhaustion" is not supported by the authors findings. The limited metabolic response seen by the authors could be caused by a number of factors such as IL-10 signalling or immature monocyte release (https://science.sciencemag.org/content/356/6337/513/tab-figures-data and https://www.nature.com/articles/s41467-018-07215-9). Neither of these possibilities are ruled out by the experiments presented in this paper.

Authors' answer: The first publication indicated by this referee (https://science.sciencemag.org/content/356/6337/513/tab-figures-data) is about bone-marrow derived macrophages, which have distinct metabolic properties if compared to monocytes. However, we quantified plasma levels of IL-10 in healthy donors (CTR) vs COVID-19 patients and found that IL-10 was higher in COVID-19 (see scatter plot below) thus ruling out the possibility that IL-10 maintains mitochondrial integrity and prevents accumulation of dysfunctional mitochondria in COVID-19 monocytes. We also ruled out the possibility that metabolic exhaustion could be due to the release of immature monocyte (that are

indeed increased in patients), as the bioenergetic performances were measured on sorted monocytes that are CD14+, at variance with immature cells. According to the International Clinical Cytometry Society the most immature monocytic cells (monoblasts and promonocyte) express bright HLA-DR but are low to negative for CD13 and CD14 expression. However, considering that we had the opportunity to quantify several chemokines and cytokines in plasma from patients and that GM-CSF was severely increased in COVID-19 patients (indicating the possible presence of emergency myelopoiesis), the presence of immature monocytes was analyzed in peripheral blood from controls and patients. The gating strategy is reported in Supplementary Figure 6 and new data in Figure 3B.

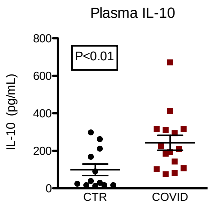

Supporting information regarding metabolic observation is practically devoid from any experimental observations made by the authors. The message of the article should be changed to match the observations made in the article.

Authors' answer: We reported data regarding oxidative phosphorylation (oxygen consumption rate) and glycolysis (extracellular acidification rate), which are important metabolic pathways in monocytes, as in other cells. However, in order to accomplish the reviewer's request, the title of the manuscript was changed as follows "Altered bioenergetics and mitochondrial dysfunction of monocytes in patients with COVID-19 pneumonia".

The authors claim that the monocytes have less ability to maintain maximal respiration; however, the maximal respiration is never the same, and the loss of looks similar to controls. This claim needs to be modified, or appropriate comparisons supporting this claim should be presented.

Authors' answer: The maintenance of maximal respiration was analyzed by calculating the area under the curve of the OCR trace from the sixth to eleventh measurement, and is different between CTR and COVID-19, as also the maximal respiration is different. However, in order to strengthen this datum, we added the analysis of the spare respiratory capacity. A scatter plot reporting the quantification of the spare respiratory capacity is now present in Figure 1A.

Claiming that classical monocytes "only tended to decrease" is not supported by statistical analysis and therefore the claim needs to reflect that.

Authors' answer: We claimed that "classical monocytes only tended to decrease" as we did not observe a statistical difference between the percentage of classical monocytes in CTR *vs* COVID-19 patients even if there was a trend and the means were slightly different among the two groups. However, as reported earlier in this point-to-point response, we applied an unsupervised approach (operator-independent approach) to analyze monocytes subpopulations in CTR and COVID-19 patients. According to this analysis classical monocytes are decreased in COVID-19 patients *vs* CTR.

I also do not understand the connection between metabolic exhaustion and PD-1/PD-L1 expression? Same to conclude on the exhausted nature of a cell because of expression of PD-1/PD-L1......expression of PD-1/PD-L1 could simply reflect the suppressive nature of the COVID-19 patient environment... and not any exhaustion. The citation Riva and Mehta states that monocyte expression of PD-1, PD-L1 and TIM-3 are involved in immune suppression, but the link to sepsis is missing. The authors need to specifically identify the source of the claim "in general, expression of inhibitory immune checkpoints, like PD-1, PD-L1 and TIM-3 has been linked to a more suppressive phenotype and worse prognosis in sepsis, infections and even cancer. If co-inhibitory receptor/ligand expression is linked to immune suppression, why is sepsis more likely in these patients?

Authors' answer: As reported before, we did not conclude on the exhausted nature of monocytes just because of expression of PD-1/PD-L1, but considering data as a whole, *i.e.* reduced OCR, reduced ECAR, reduced oxidative burst, expression of inhibitory receptors. Moreover, the expression of PD-1/PD-L1 could not simply reflect the suppressive nature of the COVID-19 patient environment as the patients' environment is both suppressive and activatory and/or inflammatory, as reported by De Biasi et al., Nat Comm (2020) and others (Mehta et al., The Lancet, 2020; Ragab et al., Front Immunol, 2020). The sources of the claim "in general, expression of inhibitory immune checkpoints, like PD-1, PD-L1 and TIM-3 has been linked to a more suppressive phenotype and worse prognosis in sepsis, infections and even cancer" are Tai et al., Am J Med Sci (2018), Zasada et al., Plos One (2017), Riva et al., Front Imm (2019), Wang et al., Immunol Invest (2017) and have been added in the bibliography.

Age and population demographics of control cohort is missing and brings into question whether the populations are comparable.

Authors' answer: Demographic information of healthy controls was not missing, but was reported in the text in the chapter "Human samples" (Materials and Methods). However, we added a column reporting demographic data of healthy controls in supplementary table 1.

Does disease severity impact monocytes differently? The authors group all covid-19 patients together, but do not show disease severity stratification.

Authors' answer: We thank the reviewer for this comment. All patients had COVID-19 pneumonia. For monocyte bioenergetics, the majority of patients (13 out of 15) were hospitalized requiring supplemental oxygen, whereas 2 out of 15 did not require supplemental oxygen. Due to the impossibility to perform any statistical analysis on two patients, these can be removed from our cohort, with no substantial changes in the results. For *in vitro* stimulation, transmission electron microscopy and plasma levels of cytokines, 15 out of 15 had COVID-19 pneumonia, were hospitalized and required supplemental oxygen.

The introduction of PD-1 expression and ligand expression is missing a citation.

Authors' answer: The citation was Xia et al., Immune Checkpoint Receptors Tim-3 and PD-1 Regulate Monocyte and T Lymphocyte Function in Septic Patients (2018). However, due to a rearrangement of the introduction, the sentence was modified.

Referee #2

In their manuscript "Metabolic exhaustion of monocytes in COVID-19 patients" the authors address COVID-19 associated changes in monocyte subset distribution, monocyte phenotype and metabolism. With their study the authors aim at elucidating mechanisms of hyper-inflammation observed in COVID-19 that are associated to monocytes. As hyper-inflammation is a complication of COVID-19 associated with severe disease and at the same time displays an attractive target for therapeutic intervention on different levels, a detailed understanding of the underlying mechanisms is urgently needed. While this is an important clinical aspect with implications also e.g. for treatment with immune checkpoint inhibitors, only very little is known about modulation of PD-1 and PDL-1 during SARS-CoV-2 infection. Therefore the Topic of the manuscript is of high novelty and potential Impact.

In their study, the authors analyzed monocyte metabolic parameters and monocyte subset distribution as well as monocyte expression of PD-1, PDL-1 and TIM-3 in COVID-19 patients and healthy controls. While novelty, medical impact and adequacy of the system are rated high, technical quality is rated medium due to lack of information on the status of the SARS-CoV-2 infection of the patients at the time-point of sampling,

i.e. in which phase of COVID-19 they were, and lack of information which of the 15 patients were analyzed for monocyte subsets and marker expression (see specific comments to the authors below).

Referee #2 (Remarks for Author):

Major comments to be addressed during revision:

1) *The authors analyzed 15 COVID-19 patients and 12 healthy controls which correspond to the data shown in figure 1. Scatter plots in figure 3 and 4 seem to show fewer data points and information on which patients were analyzed and why not all patients were analyzed is lacking.*

Authors' answer: We agree with the reviewer with this comment. As explained for referee #1, our primary endpoint was to investigate the bioenergetics of monocytes isolated from COVID-19 patients and healthy donors. We postulated that this impairment could be due to different monocyte distribution, so in a smaller cohort of patients, we analyzed monocyte phenotype.

2) While longitudinal analyses for more time-points would be optimal but are not always feasible or possible, more information on the patients' disease and inflammatory status at the time-point of analysis would facilitate integration and interpretation of the results. Relevant information would be whether these patients were altogether in a similar stage of the disease, what time-point from diagnosis/onset of the symptoms/positive test the samples were taken or whether they had already developed a specific adaptive immune/antibody response.

Authors' answer: We apologize with the reviewer for not specifying in the manuscript that the levels of D-dimer and C-reactive protein (CRP) were obtained from a blood specimen from the same day or one day after/before the day in which monocytes were analyzed. Elevated levels of D-dimer and CRP, as those observed in our cohort of patients, are strong indicator of inflammation (Bao et al, AMJ, 2017; Borowiec et al, Adv Med Sci, 2020). However, among the clinical characteristics of patients we added the neutrophil-to-lymphocyte ratio, which has been associated with disease severity and death (Liu et al., 2020; Merad et al., 2020). Neutrophil-to-lymphocyte ratio was 7.2 in COVID-19 patients vs 1.28 in CTR. This information has been added in Supplementary Table 1 and in the text. Concerning the stage of the disease, all patients had COVID-19 pneumonia. For monocyte bioenergetics, the majority of patients (13 out of 15) were hospitalized requiring supplemental oxygen, whereas 2 out of 15 were not requiring supplemental oxygen. Due to the impossibility to perform any statistical analysis on two patients, these can be removed from our cohort, with no substantial changes in the results. For in vitro stimulation, transmission electron microscopy and plasma levels of cytokines, 15 out of 15 had COVID-19 pneumonia, were hospitalized and required supplemental oxygen. New plots have been reported accordingly.

3) The interpretation of the clinical relevance of the presented findings would be greatly increased, if there are correlations to inflammatory parameters. If the authors have analyzed, or have a chance to analyze systemic cytokine levels such as for IL-6 for the time-point of monocyte analysis, this should be shown or done. As is stated also by the authors, it is a limitation that cytokine production by the monocytes themselves could not be analyzed - this is the case especially as monocyte metabolic exhaustion seems somewhat counter-intuitive in hyper-inflammation. Also therefore, any correlation to the disease and inflammatory status of the patients at the time-point of analysis would be very interesting and helpful.

Authors' answer: We have quantified a huge amount of cytokines in plasma from a total of 15 patients, and provided the data not only regarding IL-6 but also on other cytokines that have a relationship with monocytes, including GM-CSF, OPN, IFN-gamma, CCL2, PD-L1, IL-18, OPN, among others.

4) According to figure 2, total monocytes and monocyte subsets were analyzed for the expression of PDL-1, TIM-3, CXCR3, CCR2 and CD38. These data are however shown only for PD-1 and PDL-1 and described for TIM-3. The description of the TIM-3 data should include "data not shown" while CXCR3, CCR2 and CD38 analyses need not to be mentioned in the text and shown in the gating strategy in figure 2, if the results of these analyses are not shown, described or discussed later in the manuscript or supplement.

Authors' answer: We thank the review for the comments. As discussed before, we have reanalyzed data applying an unsupervised approach to detect monocyte subpopulations in CTR and COVID-19 patients. Multiparametric unsupervised method, such as the combination of different algorithms of automatic clustering (FlowSOM), dimension reduction representation (UMAP) and statistical methods for differential discovery analyses in high-dimensional cytometry data (diffcyt) are mostly automatic and operator independent. A new Figure (now Figure 2) has been added to show the results of this analysis that confirm results obtained by manual gating. The manual gating strategy and data obtained with this method have been moved to the supplementary material (Supplementary Figure 3 and 4).

Minor comments to be addressed during revision:
1) Organization of multi-panel figures into A, B etc. would facilitate reading and finding the relevant (sub)-figure.

Authors' answer: As suggested by the reviewer, we organized the figures with multi-panels.

2) Please check scatter-plots in figure 1 as Max Resp maintenance is listed in the figure legend but Basal ECAR is shown (and not listed in the legend).

Authors' answer: We checked and amended.

3) Are those few patients showing substantially higher values in the bioenergetics profile (figure 1) always the same patients and does this correlate with any other parameter of the disease (along major comments 2) and 3))?

Authors' answer: We thank the reviewer for this comment. As reported earlier, all patients had COVID-19 pneumonia. For monocyte bioenergetics, the majority of patients (13 out of 15) were hospitalized requiring supplemental oxygen, whereas 2 out of 15 were not requiring supplemental oxygen (and were those with the higher values in the bioenergetics profile). Due to the impossibility to perform any statistical analysis on two patients, these can be removed from our cohort, with no substantial changes in the results. For in vitro stimulation, transmission electron microscopy and plasma levels of cytokines, 15 out of 15 had COVID-19 pneumonia, were hospitalized and required supplemental oxygen. New plots are present in the manuscript.

4) In the gating strategy (figure 2) the description is a bit misleading regarding CD15/CD11b gating. The text states "Then, cells positive for HLA-DR and CD15 (i.e., leukocytes that did not include lymphocytes, that are CD15-) were selected" and CD11b is not referred to, while in the corresponding plot the y-axis is labelled CD15/CD11b and there does not seem to be a selection for the CD15/CD11b-positive population but rather all HLA-DR positive cells are gated.

Authors' answer: We apologize with the reviewer. This was a mistake, thus we amended the text as follows "Then, cells positive for HLA-DR, CD15 and CD11b were selected and monocytes expressing CD14 were identified."

General comments (not necessarily need to be addressed for publication but of general interest):
1) As this manuscript clearly focusses in monocytes, also T cell PD1/PD-L1 expression would be of high interest.

Authors' answer: We totally agree with the reviewer. Together with monocytes, T cell exhaustion is also very interesting. We recently published that COVID-19 patients' T cell compartment displays several

alterations involving naïve, central memory, effector memory and terminally differentiated cells, as well as regulatory T cells and PD1+CD57+ exhausted T cells (De Biasi et al, Nature Communications, 2020).

2) It would be interesting to comment or speculate on the relevance of these results on a treatment with check-point inhibitors in cancer patients (during concomitant COVID-19).

Authors' answer: This suggestion is very important and we thank the reviewer for this. We discussed this point, by adding the following paragraph "The upregulation of PD-1 and PD-L1 on monocytes from COVID-19 raises important clinical questions for the potential intersection between COVID-19 and cancer immunotherapy, being immune checkpoint inhibitors (ICI) the pillar of cancer therapy in several tumors (Maio et al, 2020). On the one hand, in the presence of the cytokine storm, ICI therapy could affect the monocytic compartment, further unbalancing the immunologic response. This, in turn, could exacerbate inflammation and therefore worsen the clinical course of COVID-19 disease. To this regard, it has been found that anti–PD-L1 therapy led to dominant gene expression changes in CD14+ monocytes (Bar et al., 2020). On the other hand, considering the T cell compartment, ICI therapy could mitigate the early phase of COVID-19 disease by contributing to viral clearance through he reactivation of potentially exhausted, PD-1+ antigen-specific T cells (Maio et al, 2020). These considerations imply that scientific evidences are now needed to provide mechanistic insights on the possible relationship between ICI and COVID-19 infection, and to clarify whether ICI could be used in cancer patients with concomitant COVID-19."

Referee #3:
This is a very interesting manuscript which characterizes functional changes in peripheral blood monocytes from patients affected by Covid 19 pneumonia. The Authors identified a redistribution among different monocytes classes accompanied by a significant expansion of intermediate/pro- inflammatory cells and an increased expression of inhibitory checkpoints, including PD-1/PD-L1. It turns out that the metabolic and functional exhaustion in monocytes from patients might alter their capability to rapidly clear the infection. The manuscript is concise, well written and unveils novel aspects of Covid 19 infection, not characterized before. I suggest that some points are addressed before publication, including experiments, if the limited material from patient samples allows to perform them:

Major points
1. In Fig. 1, left panel with representative traces, it is not clear to me if cells from Covid patients contain less mitochondria than control monocytes or if their mitochondria are simply less functional. A difference in number of mitochondria might explain a different basal and maximal respiration. Cardiolipin staining with Nonyl Acridine Orange (NAO) fluorescent mitochondrial dye whose staining is independent of mitochondrial

membrane potential, might help to characterize the mechanism causing lower respiration in cells derived from Covid patients.

Authors' answer: We thank the reviewer for this remark. We quantified mitochondrial mass by using cells mitotracker green, which stains mitochondria regardless of mitochondrial membrane potential. Data have been added in the manuscript and in Supplementary Figure 1A. Information regarding mitochondrial mass were obtained also from transmission electron microscopy and were reported in Figure 1B. Mitochondrial membrane potential was also analysed by staining cells with JC-1, and data have been collected in Supplementary Figure 1C.

2. Western blotting of respiratory complex subunits, ATP synthase and its inhibitor protein IF1 should be performed, if lysates from patient cells are available. On one hand OXPHOS detection will provide a possible explanation for the functional results presented in Fig. 1, on the other hand, the IF1 levels would be particularly interesting given the emerging role of this protein in the modulation of ATP synthase in pathological condition and inflammation (García-Aguilar A and Cuezva JM, Front Physiol 2018. Review of the Inhibition of the Mitochondrial ATP Synthase by IF1 in vivo: Reprogramming Energy Metabolism and Inducing Mitohormesis; Satapati S et al., J Clin Invest 2015. Mitochondrial metabolism mediates oxidative stress and inflammation in fatty liver).

Authors' answer: We are aware that western blotting of ETC complexes and IF1 could complete data regarding OXPHOS, thus improving the quality of the manuscript. We thank the reviewer for this comment. However, unfortunately, to obtain protein lysates from monocytes from a clinical sample is quite challenging as the number of cells required to have even a few mg of proteins is high. Monocytes are indeed 10-20% of peripheral blood mononuclear cells (PBMCs). We usually obtained twenty mL of peripheral blood from patients and controls. From healthy blood, PBMCs yield ranges between 0.5-3 million cells/mL (10-60 million cells in 20 mL). Most COVID-19 patients has leukopenia, thus they had a severe reduction in the number of leukocytes, including monocytes. Therefore, the number of cells was insufficient to get protein lysates to perform western blotting.

Minor points

1. I suggest to the Authors to briefly mention in the introduction the connection between monocyte ATP level (bioenergetic performance/glycolysis) and their role in the infection clearance. This would help the less specialized audience to follow the importance of the presented data and understand the relevance of differences in the respiratory behavior of patient cells.

Authors' answer: We thank the reviewer for this suggestion. We added the following sentence in the introduction: "Recent studies have shown that monocyte bioenergetics and inflammatory phenotype exist within a spectrum and adapt in response to different stimuli (Yamada et al, 2020). Other studies demonstrated that the microbial stimulation of different Toll-like receptors induces different metabolic programs in monocytes (Lachmandas et al, 2016). For example, monocytes after stimulation with whole-pathogen lysates from Escherichia coli, Staphylococcus aureus and M. tuberculosis, and with the TLR2 ligand Pam3CysSK4, increased OCR and glycolysis, which are essential for activation of host defence mechanisms such as cytokine production and phagocytosis. Functional and metabolic re-programming has been also observed in monocytes during sepsis in a process mediated by hypoxia-inducible factor-1α (HIF1α) (Shalova et al, 2015)."

2. In Supplementary Table 1, a range of values in normal condition (healthy donors) for bilirubin, LDH, and other parameters (that are reported in the text) should be indicated on the right in the same table, for clarity.

Authors' answer: We thank the reviewer for the suggestions. We added a column reporting values of healthy controls in Supplementary Table 1.

3. The "oxidative burst" which is stimulated by ionomycin treatment should be better explained in the text. This treatment may cause multiple effects such as Ca2+ uptake in mitochondria, stimulation of the respiratory chain, depolarization of the mitochondrial membranes, ... The Author should better explain which of these effects are in their opinion emerging in their experimental conditions and help the reader to understand the importance of differences between patient and control monocytes.

Authors' answer: We thank the reviewer for this comment as it gave us the possibility to clarify this point. We are aware that the treatment may cause effects such as Ca2+ uptake, stimulation of OXPHOS, among others. However, our purpose was to quantify the oxidative burst as an indicator of monocytic function. To further clear up this point we added the following sentence: " The respiratory or oxidative burst is the rapid release of reactive oxygen species (ROS) from different cell types, including monocytes, and its quantification is a direct measure of activation and phagocytic function (Vergis et al, 2017). The capacity to perform the oxidative burst in response to a challenge, together with phagocytosis, microbial killing and cytokine production are main functions of monocytes, and are influenced by the metabolic state of the cell (McBride et al., 2020). During the oxidative burst, a large amount of oxygen is consumed generating ROS, to kill pathogens. For this reason, to investigate the complete kinetic range of the maximal burst and the immediate response, oxidative burst was measured by calculating the AUC between the tenth and the thirteenth timepoint and by analyzing the oxygen consumption at the eleventh timepoint, respectively."

7th Oct 2020

Dear Dr. Gibellini,

Thank you for the submission of your revised manuscript to EMBO Molecular Medicine. We have now received the enclosed reports from the referees that were asked to re-assess it. As you will see the reviewers are now globally supportive and I am pleased to inform you that we will be able to accept your manuscript pending the following final amendments:

***** Reviewer's comments *****

Referee #2 (Comments on Novelty/Model System for Author):

The comments raised in the first round of review have been largely adressed and the manuscript has gained from the revisions and the newly included data and points discussed. The title of the revised manuscript better refers to the data shown. The study remains descriptive, which however should not be a problem at this stage, taking into account limited availability of material and novelty of the topic.

Referee #2 (Remarks for Author):

There are minor comments to be addressed:

#1: in the text, page 4, correct spelling of E. coli, S. aureus and M. tuberculosis
#2: readability of figure 1B would be improved if subpanels a, b, c, d and e were labelled CTR or COVID within the figure.
#3 (this is the most important point): from the materials and methods section it does not become clear, which patients within the cohort of healthy controls and COVID-19 patients went into which analyses. It makes a large difference, whether e.g. plasma cytokine levels are from the same patients in which also monocyte subsets were analyzed or whether all of the analyses (plasma cytokines, monocyte subsets, immature monocytes, in vitro cytokine production, bioenergetic profile) were performed in separate sets of patients. This should be specified.
#4: please specify in the materials and methods section out of how many patient (and control) samples the 225 mitochondria analysed for each group were derived.

Referee #2

Referee #2 (Comments on Novelty/Model System for Author):

The comments raised in the first round of review have been largely addressed and the manuscript has gained from the revisions and the newly included data and points discussed. The title of the revised manuscript better refers to the data shown. The study remains descriptive, which however should not be a problem at this stage, taking into account limited availability of material and novelty of the topic.

Referee #2 (Remarks for Author):

There are minor comments to be addressed:

#1: in the text, page 4, correct spelling of E. coli, S. aureus and M. tuberculosis

Authors' answer: We corrected spelling.

#2: readability of figure 1B would be improved if subpanels a, b, c, d and e were labelled CTR or COVID within the figure.

Authors' answer: We improved readability by adding labels in each panel.

#3 (this is the most important point): from the materials and methods section it does not become clear, which patients within the cohort of healthy controls and COVID-19 patients went into which analyses. It makes a large difference, whether e.g. plasma cytokine levels are from the same patients in which also monocyte subsets were analyzed or whether all of the analyses (plasma cytokines, monocyte subsets, immature monocytes, in vitro cytokine production, bioenergetic profile) were performed in separate sets of patients. This should be specified.

Authors' answer: We added requested information in the material and methods section. In particular we added: *"Twenty-eight patients with COVID-19 pneumonia admitted at the University Hospital in Modena (Italy) in March-July 2020, were included in this study. They had a median age of 63 years (range 37-89), 68.9% were males. Among them, a sub-cohort of thirteen patients was used to perform bioenergetics, ultrastructural analysis of mitochondria, quantification of monocyte subsets, immature monocytes and in vitro production of cytokines. A sub-cohort of fifteen patients was used to analyse plasma levels of the indicated cytokines and chemokines. Besides, 27 healthy individuals were recruited from the University Hospital personnel during the same period and served as normal controls (median age 58 years; range 35-80 years; 53.5% were males). Among them, a sub-cohort of twelve healthy donors was used to perform bioenergetics, ultrastructural analysis of mitochondria, quantification of monocyte subsets, immature monocytes and in vitro production of cytokines. A sub-cohort of fifteen of healthy controls was used to analyse plasma levels of the indicated cytokines and chemokines."*

#4: please specify in the materials and methods section out of how many patient (and control) samples the 225 mitochondria analysed for each group were derived.

Authors' answer: we specified in the materials and methods, paragraph "transmission electron microscopy", that 225 mitochondria were from three healthy controls and three COVID patients.

3rd Revision - Editorial Decision 14th Oct 2020

The authors performed the requested changes.

EMBO PRESS

YOU MUST COMPLETE ALL CELLS WITH A PINK BACKGROUND ↓

PLEASE NOTE THAT THIS CHECKLIST WILL BE PUBLISHED ALONGSIDE YOUR PAPER

Corresponding Author Name: Lara Gibellini
Journal Submitted to: EMBO Molecular Medicine
Manuscript Number: EMM-2020-13001-V3

USEFUL LINKS FOR COMPLETING THIS FORM

http://www.antibodypedia.com
http://1degreebio.org
http://www.equator-network.org/reporting-guidelines/improving-bioscience-research-report
http://grants.nih.gov/grants/olaw/olaw.htm
http://www.mrc.ac.uk/Ourresearch/Ethicsresearchguidance/Useofanimals/index.htm
http://ClinicalTrials.gov
http://www.consort-statement.org
http://www.consort-statement.org/checklists/view/32-consort/66-title
http://www.equator-network.org/reporting-guidelines/reporting-recommendations-for-tumo
http://datadryad.org
http://figshare.com
http://www.ncbi.nlm.nih.gov/gap
http://www.ebi.ac.uk/ega
http://biomodels.net/
http://biomodels.net/miriam/
http://jjj.biochem.sun.ac.za
http://oba.od.nih.gov/biosecurity/biosecurity_documents.html
http://www.selectagents.gov/

Reporting Checklist For Life Sciences Articles (Rev. June 2017)

This checklist is used to ensure good reporting standards and to improve the reproducibility of published results. These guidelines are consistent with the Principles and Guidelines for Reporting Preclinical Research issued by the NIH in 2014. Please follow the journal's authorship guidelines in preparing your manuscript.

A- Figures
1. Data
The data shown in figures should satisfy the following conditions:

- the data were obtained and processed according to the field's best practice and are presented to reflect the results of the experiments in an accurate and unbiased manner.
- figure panels include only data points, measurements or observations that can be compared to each other in a scientifically meaningful way.
- graphs include clearly labeled error bars for independent experiments and sample sizes. Unless justified, error bars should not be shown for technical replicates.
- if n< 5, the individual data points from each experiment should be plotted and any statistical test employed should be justified
- Source Data should be included to report the data underlying graphs. Please follow the guidelines set out in the author ship guidelines on Data Presentation.

2. Captions
Each figure caption should contain the following information, for each panel where they are relevant:

- a specification of the experimental system investigated (eg cell line, species name).
- the assay(s) and method(s) used to carry out the reported observations and measurements
- an explicit mention of the biological and chemical entity(ies) that are being measured.
- an explicit mention of the biological and chemical entity(ies) that are altered/varied/perturbed in a controlled manner.

- the exact sample size (n) for each experimental group/condition, given as a number, not a range;
- a description of the sample collection allowing the reader to understand whether the samples represent technical or biological replicates (including how many animals, litters, cultures, etc.).
- a statement of how many times the experiment shown was independently replicated in the laboratory.
- definitions of statistical methods and measures:
  - common tests, such as t-test (please specify whether paired vs. unpaired), simple $\chi 2$ tests, Wilcoxon and Mann-Whitney tests, can be unambiguously identified by name only, but more complex techniques should be described in the methods section;
  - are tests one-sided or two-sided?
  - are there adjustments for multiple comparisons?
  - exact statistical test results, e.g., P values = x but not P values < x;
  - definition of 'center values' as median or average;
  - definition of error bars as s.d. or s.e.m.

Any descriptions too long for the figure legend should be included in the methods section and/or with the source data.

In the pink boxes below, please ensure that the answers to the following questions are reported in the manuscript itself. Every question should be answered. If the question is not relevant to your research, please write NA (non applicable). We encourage you to include a specific subsection in the methods section for statistics, reagents, animal models and human subjects.

B- Statistics and general methods

Please fill out these boxes ↓ (Do not worry if you cannot see all your text once you press return)

1.a. How was the sample size chosen to ensure adequate power to detect a pre-specified effect size?	We studied all the patients that were recruitable during and after the lock-down period. The number of patients per set of experiments was chosen using the software available on line for clinical and lab studies, according to the probability to reach a statistical significance when compared to controls.
1.b. For animal studies, include a statement about sample size estimate even if no statistical methods were used.	NA
2. Describe inclusion/exclusion criteria if samples or animals were excluded from the analysis. Were the criteria pre-established?	We enrolled 28 patinets with pneumonia caused by COVID-19 who had been admitted to the Infectious Disease Clinics of the University Hospital in Modena, North Italy. Age range 37-89, 69% were males, all caucasians. They were compared with 27 age- and sex- martched healthy donors. Blood samples were collected at the Infetious Disease CLinics, brought to the Immunology Lab (500 meters away) and immediately processed.
3. Were any steps taken to minimize the effects of subjective bias when allocating animals/samples to treatment (e.g. randomization procedure)? If yes, please describe.	There was no randomization, being a study that compares patients with COVID pneumonia vs. age- and sex-matched healthy donors
For animal studies, include a statement about randomization even if no randomization was used.	NA
4.a. Were any steps taken to minimize the effects of subjective bias during group allocation or/and when assessing results (e.g. blinding of the investigator)? If yes please describe.	NA
4.b. For animal studies, include a statement about blinding even if no blinding was done	NA
5. For every figure, are statistical tests justified as appropriate?	Yes. Statistical analysis is reported in the Material and Methods section. P value is reported in Figure legends.
Do the data meet the assumptions of the tests (e.g., normal distribution)? Describe any methods used to assess it.	Yes. Statistical analysis is reported in the Material and Methods section. P value is reported in Figure legends.
Is there an estimate of variation within each group of data?	The estimate of variation was performed according to the data already published by others.
Is the variance similar between the groups that are being statistically compared?	All of the statistical analyses that we have performed were chosed considering the variance of the population under examination.

C- Reagents

6. To show that antibodies were profiled for use in the system under study (assay and species), provide a citation, catalog number and/or clone number, supplementary information or reference to an antibody validation profile. e.g., Antibodypedia (see link list at top right), 1DegreeBio (see link list at top right).	Yes, we provide a table (supplemenatary table 2) where the list of monoclonal antibodies and fluorochromes used for the cytofluorimetric analysis of monocytes is reported.
7. Identify the source of cell lines and report if they were recently authenticated (e.g., by STR profiling) and tested for mycoplasma contamination.	NA

* for all hyperlinks, please see the table at the top right of the document

D- Animal Models

8. Report species, strain, gender, age of animals and genetic modification status where applicable. Please detail housing and husbandry conditions and the source of animals.	NA
9. For experiments involving live vertebrates, include a statement of compliance with ethical regulations and identify the committee(s) approving the experiments.	NA
10. We recommend consulting the ARRIVE guidelines (see link list at top right) (PLoS Biol. 8(6), e1000412, 2010) to ensure that other relevant aspects of animal studies are adequately reported. See author guidelines, under 'Reporting Guidelines'. See also: NIH (see link list at top right) and MRC (see link list at top right) recommendations. Please confirm compliance.	NA

E- Human Subjects

11. Identify the committee(s) approving the study protocol.	Area Vasta Emilia Romagna, protocol number 177/2020, March 10th, 2020
12. Include a statement confirming that informed consent was obtained from all subjects and that the experiments conformed to the principles set out in the WMA Declaration of Helsinki and the Department of Health and Human Services Belmont Report.	Informed consent was obtained from all subjects and the experiements were set out in the WMA Declaration of Helsinki.
13. For publication of patient photos, include a statement confirming that consent to publish was obtained.	NA
14. Report any restrictions on the availability (and/or on the use) of human data or samples.	NA
15. Report the clinical trial registration number (at ClinicalTrials.gov or equivalent), where applicable.	NA
16. For phase II and III randomized controlled trials, please refer to the CONSORT flow diagram (see link list at top right) and submit the CONSORT checklist (see link list at top right) with your submission. See author guidelines, under 'Reporting Guidelines'. Please confirm you have submitted this list.	NA
17. For tumor marker prognostic studies, we recommend that you follow the REMARK reporting guidelines (see link list at top right). See author guidelines, under 'Reporting Guidelines'. Please confirm you have followed these guidelines.	NA

F- Data Accessibility

18: Provide a "Data Availability" section at the end of the Materials & Methods, listing the accession codes for data generated in this study and deposited in a public database (e.g. RNA-Seq data: Gene Expression Omnibus GSE39462, Proteomics data: PRIDE PXD000208 etc.) Please refer to our author guidelines for 'Data Deposition'.<br><br>Data deposition in a public repository is mandatory for:<br>a. Protein, DNA and RNA sequences<br>b. Macromolecular structures<br>c. Crystallographic data for small molecules<br>d. Functional genomics data<br>e. Proteomics and molecular interactions	NA
19. Deposition is strongly recommended for any datasets that are central and integral to the study; please consider the journal's data policy. If no structured public repository exists for a given data type, we encourage the provision of datasets in the manuscript as a Supplementary Document (see author guidelines under 'Expanded View' or in unstructured repositories such as Dryad (see link list at top right) or Figshare (see link list at top right).	The source data underlying Figure 2, 3B, Supplemenatry Figs 1-6 can be provided as a Source Data file. Original .fcs concerning cytofluorimetric analysis can be deposited at the flow repository.org
20. Access to human clinical and genomic datasets should be provided with as few restrictions as possible while respecting ethical obligations to the patients and relevant medical and legal issues. If practically possible and compatible with the individual consent agreement used in the study, such data should be deposited in one of the major public access-controlled repositories such as dbGAP (see link list at top right) or EGA (see link list at top right).	NA
21. Computational models that are central and integral to a study should be shared without restrictions and provided in a machine-readable form. The relevant accession numbers or links should be provided. When possible, standardized format (SBML, CellML) should be used instead of scripts (e.g. MATLAB). Authors are strongly encouraged to follow the MIRIAM guidelines (see link list at top right) and deposit their model in a public database such as Biomodels (see link list at top right) or JWS Online (see link list at top right). If computer source code is provided with the paper, it should be deposited in a public repository or included in supplementary information.	NA

G- Dual use research of concern

22. Could your study fall under dual use research restrictions? Please check biosecurity documents (see link list at top right) and list of select agents and toxins (APHIS/CDC) (see link list at top right). According to our biosecurity guidelines, provide a statement only if it could.	NA
